# Community Care Workers, Poor Referral Networks and Consumption of Personal Resources in Rural South Africa

**DOI:** 10.1371/journal.pone.0095324

**Published:** 2014-04-29

**Authors:** Ilona Sips, Ahmad Haeri Mazanderani, Helen Schneider, Minrie Greeff, Francoise Barten, Mosa Moshabela

**Affiliations:** 1 Radboud UMC, Department of Primary and Community Care, International Health, Nijmegen International Center for Health Systems Research and Education (NICHE), Nijmegen, The Netherlands; 2 University of Pretoria, Department of Medical Virology, Pretoria, South Africa; 3 National Health Laboratory Services, Tshwane Academic Division, Pretoria, South Africa; 4 University of Western Cape, School of Public health, Cape Town, South Africa; 5 North–West University, Africa Unit for Transdisciplinary Health Research, Faculty of Health Science, Potchefstroom, South Africa; 6 University of Witwatersrand, School of Public Health, Johannesburg, South Africa; 7 Earth Institute, Columbia University, New York City, United States of America; University of Oxford, Kenya

## Abstract

Although home-based care (HBC) programs are widely implemented throughout Africa, their success depends on the existence of an enabling environment, including a referral system and supply of essential commodities. The objective of this study was to explore the current state of client referral patterns and practices by community care workers (CCWs), in an evolving environment of one rural South African sub-district. Using a participant triangulation approach, in-depth qualitative interviews were conducted with 17 CCWs, 32 HBC clients and 32 primary caregivers (PCGs). An open-ended interview guide was used for data collection. Participants were selected from comprehensive lists of CCWs and their clients, using a diversified criterion-based sampling method. Three independent researchers coded three sets of data – CCWs, Clients and PCGs, for referral patterns and practices of CCWs. Referrals from clinics and hospitals to HBC occurred infrequently, as only eight (25%) of the 32 clients interviewed were formally referred. Community care workers showed high levels of commitment and personal investment in supporting their clients to use the formal health care system. They went to the extent of using their own personal resources. Seven CCWs used their own money to ensure client access to clinics, and eight gave their own food to ensure treatment adherence. Community care workers are essential in linking clients to clinics and hospitals and to promote the appropriate use of medical services, although this effort frequently necessitated consumption of their own personal resources. Therefore, risk protection strategies are urgently needed so as to ensure sustainability of the current work performed by HBC organizations and the CCW volunteers.

## Introduction

South Africa's health care system, like other low and middle income countries, struggles to cope with the collision of four excessive health burdens: (1) communicable diseases, especially HIV/AIDS and Tuberculosis (TB); (2) non-communicable diseases; (3) maternal, neonatal and child deaths and; (4) deaths from injury and violence and underlying determinants [1], [2]. The deficiencies in the health system are caused by a combination of factors, including South Africa's colonial and apartheid history [3], inefficient government spending, shortage of material and human resources, especially in rural areas, and lack of clear policy reform [4]. These difficulties mount additional pressure on the already vulnerable and weak health care system [5], [6], with a particular negative impact in rural areas [7]. As a consequence, community members have increasingly adopted ‘caring’ roles in places where services are inadequate or unavailable [8]. The principal responsibility of providing care for people with chronic conditions (including HIV/AIDS) has subsequently shifted from the health care system to local communities and households [9]. Care at home can potentially alleviate many of the unmet primary health care needs in the developing world, including reducing health inequalities of marginalized and difficult to reach rural communities [10].

From the late 1990's, non-governmental organizations and faith based ‘home-based care’ (HBC) projects began to increase across the country, using lay workers to provide palliative care to HIV infected people. Although HBC was originally introduced to provide palliative care to people with HIV/AIDS outside of the hospital setting, it has been evolving into a more general health and social support sector, and a safety net for vulnerable individuals and households. In 2000, the South African national government began to allocate ring-fenced grants to strengthen home and community based care projects, leading to a rapid growth of state-supported non-profit organizations employing community care givers [8].

In order for community care worker programs to be successful Celletti et al. [11] have identified, amongst other factors, the need for adequate systems integration, a functioning referral system, mentoring and supervision, and collaborative planning. Furthermore, the recognition of roles, skills and contributions made by community care workers (CCWs) is considered essential for the success of HBC, and effectively links these workers into the existing health care system.

Community health programs have a variety of typologies, all of which consist of their own set of services, delivery schemes, staff and areas of service provision. A distinction needs to be made between HBC and community health worker programs in South Africa, as their emphasis is different. Community home-based care programs focus on psychosocial and household support for clients and their families provided through a volunteer network in the community. Alternatively, community health worker programs focus on the medical aspects of care provision, provided by a team involving health care professionals [12]. Home-based care programs are operated by CCWs, also referred to as home-based carers, who are a heterogeneous group of volunteers, providing HBC and additional services that are poorly defined under the auspices of a HBC organization. Community health worker programs comprise a new cadre of paid workers within the South African Department of Health, who are trained and have well-defined roles [8].

By stimulating social action, CCWs can encourage community participation in the health system and political environment [13], [14]. Hence, they can potentially enable a community-based sector of health care and social support that does not substitute, but rather complements the more specialized services of professional cadres within the health care system [15]. The development of HBC organizations in urban areas of South Africa, regardless of whether they are funded by the Department of Health or not, is far from complete [20]. The situation is likely to be worse in rural areas, compared to urban centres, given the nature of health care inequities in South Africa, which favor the latter [5]. Furthermore, substantial investments in strengthening the relationship between HBC and the health system are still needed to boost social capital at community level and enable CCWs to effectively fight the HIV/AIDS and TB co-epidemics [16]. In order to inform policy processes in the country, we conducted a large research project to investigate the quality of care provided by the CCWs in the home. In the context of this study, we sought to investigate the current state of CCW referral patterns and practices, between their clients' homes and the health care system.

## Methods

### Study design

We conducted an in-depth qualitative study with three groups of participants using a triangulation method to form triads of CCW-Client-Caregiver. In the triad, the first was a CCW, who is in charge of providing services to the client in the home. The second was a client who received care in the home environment. Finally, the primary care giver (PCG) or main family caregiver completed the triad. These PCGs are individuals who provide care and support to the client around the clock within the home setting. In this way, triangulation allowed for a convergence of perspectives from three different participants in a home caring environment, and hence used to increase the trustworthiness of our findings [17]. The current study formed part of a larger three-year project conducted in Bushbuckridge, South Africa that investigated the quality of care and support provided to care recipients in their homes and their respective primary caregivers.

### Study setting

Bushbuckridge is a rural municipality located in Mpumalanga province in the north-east of South Africa, covering an area of 2580 km^2^, with a population of over 500 000 people [18]. Bushbuckridge is served by three hospitals, two community health centers, 34 clinics and two mobile clinics. The area is designated as one of the 22 most poverty-stricken areas in South Africa, characterized by underdevelopment, high unemployment rates, and poor service delivery. Although Bushbuckridge borders the Kruger National Park, and harbors a number of private game and nature reserves, there is limited involvement and benefits in tourism for the local communities [18]. The HIV/AIDS prevalence, which is significantly higher than the national average (35,1% vs. 30,2% HIV prevalence among antenatal clinic attendees) [19], results in a high nurse workload, found to be 50% higher than the South African average at district level in 2006 [20]. In 2010, at the time of data collection, there were 943 CCWs in Bushbuckridge caring for sick people in their homes through 37 HBC organizations.

### Study population and sampling

Home-based care organizations in Bushbuckridge were identified through existing records from the Departments of Health and Social Development, and a snowball sampling technique was used to identify unregistered HBC organizations [21]. Of the 37 identified organizations, a subset of nine was selected using a criterion-based sampling method as indicated in Table 1. This was intended to ensure the inclusion of a wide range of HBC organizations. From each of the nine selected HBC organizations, two CCWs were selected using a stratified criterion-based sampling method. From a list of clients for all selected CCWs, two client participants were selected. Once again, a diversified criterion was used to ensure a wide range of representation among the client population. All selected clients were asked to identify their PCG, who was included in the study if the participant was at least 18 years old. As a result, a total of 17 CCWs, 32 clients and their 32 PCGs, all adults, were included in this study.

**Table 1:** Criteria used for sampling of HBC organizations, CCWs, clients and their PCGs.

Participants	Sampling Criteria
*Home based care organizations*	a) Supervision of the CCW/availability of CCW coordinator; b) Whether the CCW have followed HBC training courses; c) Whether the HBC has regular de-briefings with their CCWs; d) How CCWs are recruited by the HBC; e) Patient –carers ratio; f) Geographical location of the HBC; g) Whether the HBC has a building (either owned or rented), or not; h) If the HBC is registered and when they got registered; i) Whether the CCW of the HBC get a stipend or not; j) Whether the HBC is funded or not; k) Community relationships with the HBC, e.g. relationship between the HBC and clinic/hospital
*Community care workers*	a) Gender; b) Age; c) Education level; d) Village they work in; e) How long they have been working as a carer; f) How long they have been working for the organization; g) Whether the CCW gets a stipend or is a volunteer; h) Which type of care the CCW provide; i) Type of patients the CCW takes care of; j) The number of patients the CCW has.
*Clients*	a) Gender; b) Age; c) Educational level; d)Village of residence; e)Disease; f)Type of care provided

### Data collection

Data collection occurred between April and December 2010 by means of face-to-face, open-ended, in-depth interviews using a topic guide. The topic guides were designed in English and translated into XiTsonga, SiSwati and SePedi. These were discussed, piloted and adapted prior to adoption in the study [22]. Three interview teams, a researcher paired with a trained field worker, performed all the interviews in the preferred language of the participant.

A subset of twenty-three follow-up interviews (eight CCWs, seven PCGs, and eight clients) was conducted with eight of the nine selected HBC organizations. Inclusion in the follow-up was intended to ensure completeness and provide clarification of flagged questions arising from initial analysis of the primary interviews, but this process was limited by time and logistical constraints. In order to guide the follow-up interviews, a tailored vignette was created for each participant to facilitate the follow-up interview. Additional questions were included if information was missing or unclear from the primary interview. One field worker team conducted all follow-up interviews. All interviews were recorded, translated and transcribed verbatim. A different researcher listened to the translations and transcription for validation purposes. Three researchers conducted quality checks independently, and coded the interviews in an inductive manner for referral practices and consumption of personal resources.

### Data analysis

An interpretive qualitative approach [23] was used to describe the phenomenon of linkage in the services offered by CCWs between the homes and the formal health care system. We sought to identify relationships and patterns of linkage rather than simply describing the phenomenon. Analysis of interviews used both manual coding and QSR International's NVivo 9 software. Data analysis was performed in a two-step process. Referral practices were first analyzed at the individual level, and in the second step, coherence and consistency within the CCW-client-PCG triads were examined. Three researchers coded the data and generated inductive codes from the empirical data, and a quality assessor validated thematic codes against the original data and between coders. Areas of inconsistencies or lacking in agreement were resolved by consensus, and using the primary empirical data. Data presented in this study relates to both description and interpretation of linkages in referral of CCW clients for health care, as well as related patterns and practices.

### Ethics statement

Written informed consent was obtained from all participants before the start of the interview. The University of Witwatersrand's Committee for Research on Human Subjects, as well as the Mpumalanga Provincial Health Research and Ethics Committee, granted ethical clearance to conduct the study.

## Results

### Description of referral patterns and practices

**Figure 1:**
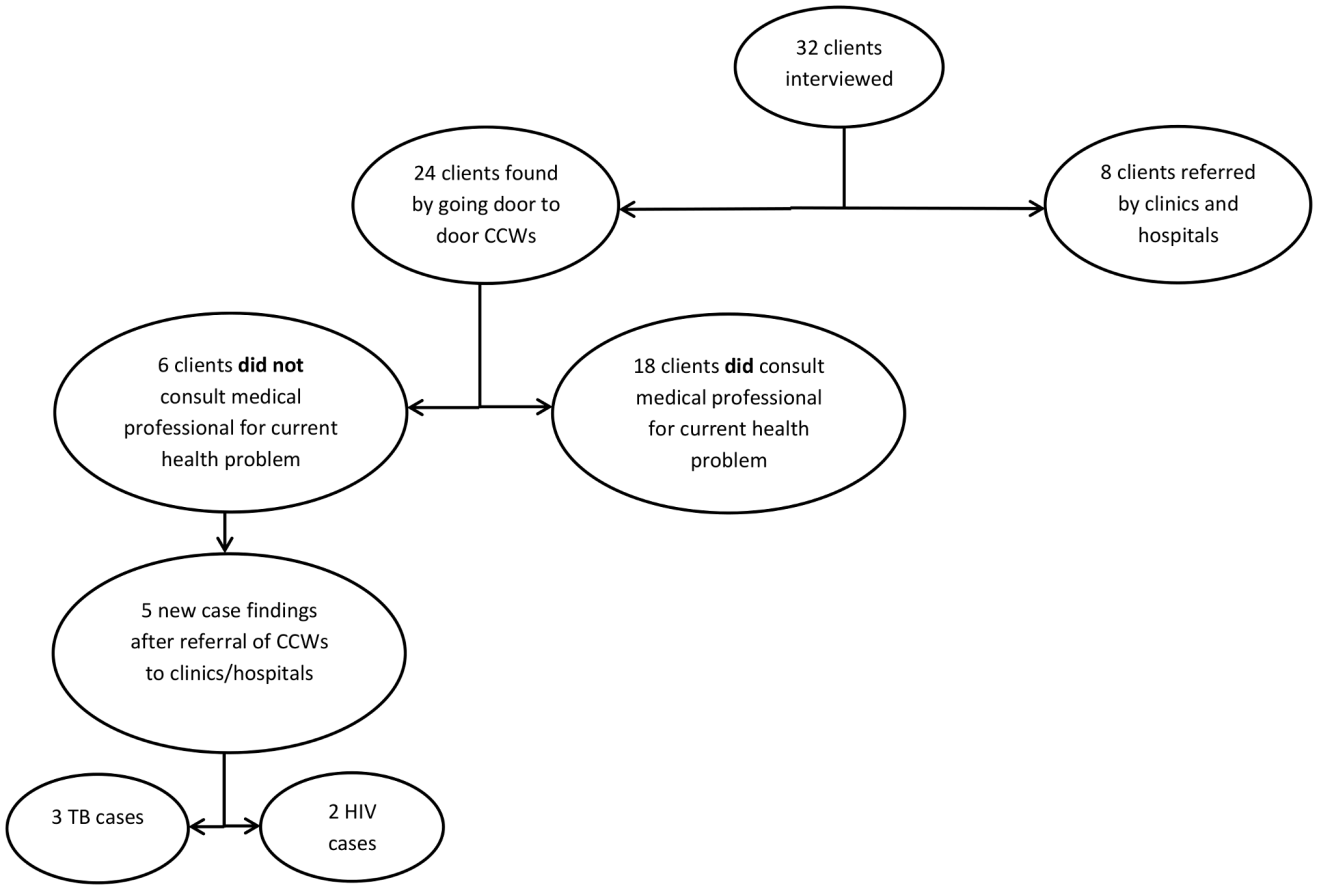
Referral practices between the health care system and home-based care programs. This figure shows client enrollment in HBC programs, showing the low referral rate of patients from health care facilities to HBC programs, especially in comparison to the total number of clients receiving medical care and their potential need of HBC.

A total of 24 of the 32 clients in this home-based care study were found by CCWs through door-to-door visits of households in the community, as they delivered health talks and searched for potential clients. The remaining eight clients were referred by a health care facility to a home-based care program. In general, there were more referrals from CCWs to health care facilities than there were from health care facilities to CCW programs in this study, as shown in Figure 1. Of the 24 clients found by CCWs in the community, 18 (3/4) had already received care from a health care facility prior to their encounter with the CCW for their current health condition(s). The remaining six clients (1/4) had not sought medical care from a health care facility, and hence their current health problem(s) had not received attention from a health care professional prior to their encounter with the CCW. In this study, three new cases of TB and two of HIV/AIDS were found among clients who had not received care from the health care facilities.

### Formation of linkages between homes and the health care system

Community care workers in this study created a bridge between homes of clients and the health facilities, and actively encouraged clients to use health care. Clients in this study, as was the case within the triad of care for client 15 who was diagnosed with TB, received their first plausible explanations for their symptoms from CCWs. These explanations were at times offered in the context of beliefs about traditional medical conditions, as indicated below.

*“Client 15 first got sick with TB and the TB was not recognized in the beginning because she had many symptoms and signs, which lead the family to believe that it was ‘mafularha’* [a traditional illness]. CCW 7, about client 15, TB

The clients themselves confirmed the relative importance of receiving support from CCWs, not only in terms of explaining medical symptoms, but also on where and how to access health care services for their medical conditions. As the client below stated, CCWs are considered to have some expertise on the health care system.

*“Her [CCW 7] role is that, as we are in our homes not knowing where we can receive care when we are sick, she is the one who knows about health care, will advise us to go to the clinic, teach us what to eat, and advise us with many things about our health”* Client 15, TB

The CCWs also made an impression on the family caregivers of the clients, who also play an important role in the health seeking behavior of the clients. The primary caregiver of client 15 recognized the role played by the CCW in rechanneling a client with TB from a traditional healer consultation to a clinic. The CCW personally accompanied this family to the clinic.

*“She [CCW 7] contributed so much. She could gather us and advise us. As a family we had already decided to take her [client 15] to the traditional doctors, but she advised us not to consider them, and she took us to the nearest clinic”* PCG 15 of client 15, TB

### Support for retention of clients in the health care system

Beyond the link established between the clients in this study and the health care system, CCWs supported adherence and retention in care. In most cases, the support was offered directly to clients and their families in the form of frequent home visits for motivational purposes.

*“When they [clients] are given another day to consult, I [CCW] visit the client a day before the date of consulting and remind the client about the consulting day. The following day I visit him to ask more and find out if he really went to the clinic. I realize that the more I keep visiting such clients, I'm encouraging them not to give up”* CCW 17

Some CCWs had to, on a regular basis, physically accompany clients, organize transport to the health facility or advocate for clients with health care providers. The success of the advocacy role played by CCWs depended on the strength of their relationships with health care providers. Therefore, they tapped into their own networks to secure help for their clients.

*“I am very much familiar with the nurses working in the clinic and they used to call me, and I would have to go there to meet clients.”* CCW 17

Community care workers had to overcome barriers within the health system as they embarked on the process of building such relationships, and often felt the need to correct any misunderstanding health professionals may hold about their roles. In this way, they made active efforts to cultivate environments through which to support their clients.

*“It was difficult in the beginning because they [health workers] didn't understand what a home-based carer did. Currently, they do understand what we are doing, and we normally have meetings with them. They also give us advice and tell us to come when we need something to help the patients”* CCW 3

It should be noted that some CCWs were not as successful in forming these relationships, and a few of them quit their work as CCWs due to such barriers. Other barriers faced by CCWs originated from the personal circumstances of the clients. In the case of CCW 9, she expressed feelings of helplessness and limitations in her ability to assist the clients.

*“Our client's [client 21] situation is not good and as we are also not earning there is nothing I can do. All we do is to give psychological support and nothing more. Sometimes we visit her and find that she doesn't have food in her house or money to go to the clinic to collect her treatment. That makes us feel sad but there is nothing we can do at this point”* CCW 9, about client 21, HIV and herpes zoster

### Use of personal resources by CCWs to support clients

While some CCWs felt helpless when their client's needs were beyond their capacity, other CCWs went as far as using their own personal resources, such as money, to ensure that their clients would visit a health care professional when required. Four CCWs made explicit reference to using their money to ensure client transportation to clinics or hospitals.

*“That little amount [stipend] that we [CCWs] are receiving, we share with the clients because when the client does not have money to go to the clinic you have to give it to him/her”* CCW 17

Community care workers also made explicit reference for the need to give their own food to ensure treatment adherence. Six CCWs stated that they share their food with their clients, sometimes doing so secretly.

*“For some I [CCW] even cook a meal in my house secretly and give to them. As you know it is not all the time where my husband will approve of me taking food from our house and giving it to my clients”* CCW 10

In general, CCWs provided resources not because they considered it their role necessarily, but because they felt compelled by the need to ensure treatment adherence or regular attendance to clinic appointments.

*“Just like when I [CCW] find a patient who isn't working I sometimes have to sacrifice myself and use my own money to transport him to the hospital or clinic. And the organization doesn't reimburse us for it nor give us a traveling allowance. Another sacrifice we make is that when we find that the patient doesn't have any food we take our own maize meal to cook for him so he can eat before taking his pills because most pills nowadays require a person to eat before taking them. He can't take them before eating. It's like we are the owners of the work and not the organization because they don't give us food parcels to give to those who don't have anything. We have to dig our own pockets”* CCW 15

These CCWs were therefore aware of the personal sacrifices they made, and the lack of compensation for it, and yet they carried on with their efforts to support and care for their clients. Furthermore, some family members of the clients, as was suggested by PCG 15 above, were aware of the efforts made by the CCW.

## Discussion

Findings of this study shed some light on the nuances of referral between CCWs and the health care system, and in particular, highlight a number deficiencies and opportunities to further strengthen this relationship for the benefit of clients enrolled in home-based care. Firstly, CCWs in our study demonstrated a high level of commitment in their effort to optimize case finding, client referral, retention and adherence of clients in the health care system. We noted that in instances where access barriers exist, CCWs repeatedly visited clients, personally escorted them, collected medicines on their behalf, and advocated for them with either family members or health providers. Secondly, we observed that the success of referral and retention in care depended on the nature of relationships between CCWs and health care providers within the health facilities. Community care workers went to great lengths in establishing and maintaining these relationships. Lastly, as evidence for their personal investment, the already vulnerable CCWs went to the extent of using their own personal resources.

The use of personal resources by CCWs, in the form of either money or food supplies, is a key theme in the results of this study. As a means of improving treatment adherence and clinic attendance, CCWs offset the costs of transport and food for clients. Since CCWs belong to the same impoverished communities they serve, these practices highlights possible exposure of CCWs to economic risk through their day-to-day work in the well-intentioned notion of service [24]. These study results suggest that CCWs may partially carry the burden of mitigating poverty when it manifests as a barrier to health care. Failure for clients to use formal care when needed could result in use of alternative health care sectors (e.g. traditional and religious healers), an increased risk of spreading infectious diseases, delayed access to appropriate care and worsened health outcomes [25]. In a previous study, the leading cause of failure to follow-up at an antiretroviral clinic in South Africa was found to be due to financial reasons, which included transport costs [26]. Similarly, lack of food to eat before taking medication and inability to pay for transportation to clinics are known causes of defaulting TB treatment [16]. In agreement with previous studies [27], [28], CCWs have a major influence on treatment adherence and clinic visits. Alarmingly, our findings suggest that such facilitation is partly established through the use of private resources of CCWs.

What is important to take into account is that, on the one hand, CCWs represent a vulnerable group of community members who are usually female, unpaid and poor, and have an increased risk of acquiring HIV and TB, and being the victims of violence [29]. Donating their personal resources to clients might increase their own risk of financial insecurity and deepening poverty. On the other hand, the provision of these resources is crucial to public health. Current CCW practices enable clients to reach the clinic, thereby potentially increasing uptake of services for HIV and TB. Whether this situation arises from a failure in the medical or social system, or a weakness in CCW programs for failing to protect their CCWs, it has to be acknowledged that CCWs compensate for the gaps in the health system by investing their own personal resources in the health and livelihood of their clients. Community care workers are expected ‘bridge’ the gap in service delivery and serve to strengthen the interface between the community and the health care system [30], [31]. However, the findings of this study necessitate that policy interventions take into account the hidden role of CCWs in building linkages that enable clients to enter and remain in the health care system.

The lack of established referral procedures and feedback mechanisms between home-based care and the health care system has been noted in this study and elsewhere [30], [32]. A study performed by Moetlo et al. [33] in Vhembe District, Limpopo Province, South Africa, found that absence of a proper referral system is a wider problem within South Africa, which should be urgently addressed in the effort to strengthen health systems. Community care workers can only fulfill their role of linking clients with formal health care if they are well-integrated into, and accepted by, a country's health care system [34], [35]. For these linkages to be effective and successful, the relationship between CCWs and providers in the health system should be characterized by mutual respect and understanding [11]. Professional health workers need to support the role of CCWs, and recognize that CCW services will allow professionals to concentrate on performing the more complicated tasks they are trained for [36], [37].

Inasmuch as the importance of linkages between home-based care and the health care system is widely acknowledged [12], [38], [39], current evidence, albeit limited, indicates that success in this regard is yet to be realized at the grass roots level [40]. Essentially, this form of informal work should be made visible to governments and policy makers not only to adequately capture the benefits, but also to enable appropriate remuneration of associated costs – financial, material and otherwise, so that the unpaid contributions can be compensated [41]. As the Department of Health in South Africa embarks on an ambitious health reform to strengthen primary health care, the country aims to implement community-based primary health care outreach teams, which will include, amongst other health care professionals, four to five community health workers per ward. It is envisaged that this initiative will attempt to incorporate CCWs into the formal health care system. The extent to which this health reform will address some of the key areas of concern in relation to HBC and CCWs remains to be determined [42]. However, one perspective is crystal clear: risk protection strategies are urgently needed to safeguard social capital of brokerage related to HBC and the wellbeing of CCWs in resource-poor settings.

### Limitations

While the ability to generalize the findings of this research is limited by its qualitative design, a limited number of interviews, and localized setting in rural South Africa, we present novel findings using a unique triangulated view of data sources in home-based care. However, views of professional health care personnel in clinics and hospitals were not included. Furthermore, comments made by the CCWs may reflect their financial interest in encouraging HBC programs to integrate with the clinics and hospitals. Lastly, quantitative methods could be used in the future to better describe patterns of case finding by CCWs in case finding, and longitudinal study designs may add richness to the exploration of referral patterns.

## Conclusions

Community care workers are not only important in linking clients to clinics and hospitals, but are also committed to promoting appropriate use of medical services. Our findings suggest there is a pervasive need to provide food and transport money to ensure adherence to clinic visits and treatment schedules. This need is frequently met by the CCWs themselves. If CCWs are not adequately remunerated for the valuable work they do, and if they continue to link clients to the formal health system through consumption of their own limited resources, they may endanger themselves, their families and their communities. In so doing they threaten the social capital they are well positioned to build within their communities, and risk entering a downward spiral of poverty and destitution.
